# Supplementary material for: Characterization of the carbapenem-resistant Acinetobacter baumannii clinical reference isolate BAL062 (CC2:KL58:OCL1): resistance properties and capsular polysaccharide structure
Source: mSystems. 2024 Sep 10;9(10):e00941-24. doi: 10.1128/msystems.00941-24 (PMC11494974; doi:10.1128/msystems.00941-24)
Supplement: Table S1 — A. baumannii genomes that carry the KL58 sequence at the K locus. [file msystems.00941-24-s0001.docx]

**Table S1.** ***A. baumannii* genomes that carry the KL58 sequence at the K locus**

| **Strain** | **NCBI accession number** | **CC/GC^1^** | **ST-IP^2^** | **Country^3^** | **Year** | **Source** |
| --- | --- | --- | --- | --- | --- | --- |
| 354_n | ERR263728 | GC2 | ST2 | Vietnam | 2006 | Carriage |
| UV_1897 | ERR197570 | GC2 | ST2 | Vietnam | 2007 | VAP^5^ |
| BAL056 | ERR190414 | GC2 | ST2 | Vietnam | 2009 | VAP |
| BAL062 | GCA_900088705.1 | GC2 | ST1550 | Vietnam | 2009 | VAP |
| BAL064 | ERR190417 | GC2 | ST2 | Vietnam | 2009 | VAP |
| BAL084 | ERR190418 | GC2 | ST2 | Vietnam | 2009 | VAP |
| BAL114 | ERR190423 | GC2 | ST2 | Vietnam | 2009 | VAP |
| BAL128 | ERR190425 | GC2 | ST2 | Vietnam | 2010 | VAP |
| BAL215 | ERR190448 | GC2 | ST2 | Vietnam | 2010 | VAP |
| BAL219 | ERR190450 | GC2 | ST2 | Vietnam | 2010 | VAP |
| BAL225 | ERR190452 | GC2 | ST2 | Vietnam | 2010 | VAP |
| BAL230 | ERR190454 | GC2 | ST2 | Vietnam | 2010 | VAP |
| BAL238 | ERR190457 | GC2 | ST2 | Vietnam | 2010 | VAP |
| BAL295 | ERR190473 | GC2 | ST2 | Vietnam | 2011 | VAP |
| BAL298 | ERR190474 | GC2 | ST2 | Vietnam | 2011 | VAP |
| BAL315 | ERR190478 | GC2 | ST2 | Vietnam | 2011 | VAP |
| BAL341 | ERR190490 | GC2 | ST2 | Vietnam | 2011 | VAP |
| BAL346 | ERR190491 | GC2 | ST2 | Vietnam | 2011 | VAP |
| BAL350 | ERR190493 | GC2 | ST2 | Vietnam | 2011 | VAP |
| BAL339 | ERR190488 | GC2 | ST571 | Vietnam | 2011 | VAP |
| BAL369 | ERR190498 | GC2 | ST2 | Vietnam | 2012 | VAP |
| BAL372 | ERR190499 | GC2 | ST2 | Vietnam | 2012 | VAP |
| BAL377 | ERR190500 | GC2 | ST2 | Vietnam | 2012 | VAP |
| BAL383 | ERR190501 | GC2 | ST2 | Vietnam | 2012 | VAP |
| UV_1268 | ERR197569 | CC10 | ST575 | Vietnam | 2005 | VAP |
| 277_ax | ERR197637 | CC10 | ST575 | Vietnam | 2005 | Carriage |
| 316_an | ERR263722 | CC10 | ST575 | Vietnam | 2006 | Carriage |
| 341_c | ERR263725 | CC10 | ST575 | Vietnam | 2006 | Carriage |
| 344_an | ERR263726 | CC10 | ST575 | Vietnam | 2006 | Carriage |
| 350_n | ERR263727 | CC10 | ST575 | Vietnam | 2006 | Carriage |
| SH37 | GCA_003070935.1 | CC10 | ST575 | China | 2010 | - |
| AB179-VUB | GCA_022459075.1 | CC10 | ST10 | Belgium | 2017 | Sputum |
| Ab-69 | GCA_027086195.1 | CC10 | ST10 | China: Hangzhou | 2020 | Urine |
| MRSN7782 | GCA_016525395.2 |  | ST150 | USA: Washington, DC | 2006 | Wound |
| MRSN31468 | GCA_006491875.1 |  | ST154 | Germany | 2003 | Groin |
| MRSN1339 | GCA_016538425.2 |  | ST154 | USA: Washington, DC | 2010 | Surveillance |
| 2021CK-01311 | GCA_020040005.2 |  | ST154 | USA | 2021 | Rectal swab |
| 2022JQ-00544 | GCA_025806895.2 |  | ST154 | USA | 2022 | Blood |
| 2023EL-00144 | GCA_029033725.2 |  | ST154 | USA | 2023 | Blood |
| 2023EL-00143 | GCA_029035645.2 |  | ST154 | USA | 2023 | catheter tip |
| 29D2 | GCA_029582015.1 |  | ST309 | Poland | 2014 | White stork nestling |
| 17-Lo_4-1 | GCA_028443315.1 |  | ST309 | Germany: Loburg | 2017 | Earthworm |
| WU_MDCI_Ab184 | GCA_025406745.1 |  | ST309 | USA: St. Louis | 2018 | respiratory tract |
| LWSM-0248 | GCA_034578555.1 |  | ST309 | Germany | 2019/2020 | Pig production settings |
| PUMA0123 | GCA_033102815.1 |  | ST309 | Singapore: Nanyang Technological University | 2023 | - |
| 16-Klo_64-1 | GCA_028444235.1 |  | ST858^4^ | Poland: Oder | 2016 | White stork |
| 98_E23.3 | GCA_027257795.1 |  | ST858^4^ | Germany | 2019 | boot swab |
| PUMA0214 | GCA_033903205.1 |  | ST342 | Singapore: Nanyang Technological University | 2023 | - |
| PUMA0184 | GCA_033102435.1 |  | ST1220 | Singapore: Nanyang Technological University | 2023 | - |
| IHIT35900 | GCA_022934425.1 |  | ST1301 | USA | 2014 | Lizard faeces |
| PUMA0099 | GCA_030554115.1 |  | ST2373 | Singapore: Nanyang Technological University | 2023 | - |
| Ab182 | GCA_018863375.1 |  | ST2561 | USA: South Carolina | 2018 | Urine |
| PUMA0145 | GCA_033103195.1 |  | ST2561 | Singapore: Nanyang Technological University | 2023 | - |
| MST-SNC-9 | GCA_032940395.1 |  | ST2643 | Canada: Ottawa | - | Agricultural surface water |
| WU_MDCI_Ab140 | GCA_025406555.1 |  | NT | USA: St. Louis | 2018 | Connective tissue infection |
| PUMA0140 | GCA_033102555.1 |  | NT | Singapore: Nanyang Technological University | 2023 | - |
| Ab14 | GCA_947045075.1 |  | NT | - | - | - |
| Ab9 | GCA_947045065.1 |  | NT | - | - | - |
| Ab11 | GCA_947044935.1 |  | NT | - | - | - |

^1^ Major clonal complex. Includes single locus variants (SLVs) and double locus variants (DLVs)

^2^ NT = not typeable

^3^ ‘-‘ = unknown

^4^ Single locus variant of ST309

^5^ VAP = Ventilator associated pneumonia
